# Intestinal Microbes in Patients With Schizophrenia Undergoing Short-Term Treatment: Core Species Identification Based on Co-Occurrence Networks and Regression Analysis

**DOI:** 10.3389/fmicb.2022.909729

**Published:** 2022-06-17

**Authors:** Min Xiang, Liqin Zheng, Daoshen Pu, Feng Lin, Xiaodong Ma, Huiqian Ye, Daoqiong Pu, Ying Zhang, Dong Wang, Xiaoli Wang, Kaiqing Zou, Linqi Chen, Yong Zhang, Zhanjiang Sun, Tao Zhang, Guolin Wu

**Affiliations:** ^1^Medical Laboratory, The Fourth People's Hospital of Ya'an, Ya'an, China; ^2^MOE Key Lab for Neuroinformation, High-Field Magnetic Resonance Brain Imaging Key Laboratory of Sichuan Province, University of Electronic Science and Technology of China, Chengdu, China; ^3^The Fourth People's Hospital of Ya'an, Ya'an, China; ^4^Psychiatry Department, The Fourth People's Hospital of Ya'an, Ya'an, China; ^5^Internal Medicine, The Fourth People's Hospital of Ya'an, Ya'an, China; ^6^The Outpatient Department, The Fourth People's Hospital of Ya'an, Ya'an, China

**Keywords:** schizophrenia, 16S rRNA sequencing, gut-brain axis, gut microbiota, fecal microbiota transplantation

## Abstract

Schizophrenia, a common mental disorder, has a tremendous impact on the health and economy of people worldwide. Evidence suggests that the microbial-gut-brain axis is an important pathway for the interaction between the gut microbiome and the development of schizophrenia. What is not clear is how changes in the gut microbiota composition and structure during antipsychotic treatment improve the symptoms of schizophrenia. In this study, 25 patients with schizophrenia were recruited. Their fecal samples were collected before and after hospital treatment for 14–19 days. The composition and structure of the intestinal microbiota were evaluated by 16S rRNA sequencing analysis, and the results showed significant differences in fecal microbiota before and after treatment. *Firmicutes* (relative abundances of 82.60 and 86.64%) and *Gemminger* (relative abundances of 14.17 and 13.57%) were the first dominant species at the phylum and genus levels, respectively. The random forest algorithm and co-occurrence network analysis demonstrated that intestinal flora (especially the core species ASV57) could be used as biomarkers to distinguish different clinical states and match treatment regimens accordingly. In addition, after fecal microbiota transplantation, antibiotic-treated recipient mice showed multiple behavioral improvements. These included decreased psychomotor hyperactivity, increased social interaction, and memory. In conclusion, this study suggests that differences in the composition and structure of gut microbiota after treatment are associated with the development and severity of schizophrenia. Results may provide a potential target for the treatment of this disorder.

## Introduction

Schizophrenia (SCZ) is a common and severe chronic mental disorder, which is manifested as hallucinations, delusions, and abnormal thoughts and behaviors. In addition, apathy and social interaction disorders are also major symptoms (Kahn et al., 2015; Xu et al., 2018). Approximately, 0.5–1% of the population suffers from SCZ globally (Yang et al., 2019), with young adults prone to higher recurrence, disability, and suicide rates (Cacabelos and Martínez-Bouza, 2011; Perez et al., 2021). Due to hallucinations and delusions, patients with SCZ are often unable to distinguish true information from their thoughts accurately. This increases their cognitive burden and leads to a high propensity for violence toward others (Tesli et al., 2020; Tikàsz et al., 2020). Suffering from chronic social interaction disorders, patients with SCZ struggle to maintain a normal life and work more than healthy individuals. Previous studies have indicated that patients with SCZ have a higher unemployment rate (Strassnig et al., 2017) and a shorter life expectancy (Stogios et al., 2021).

Schizophrenia has become a serious public health issue and social problem due to the unknown pathogenesis of the disease, the seriousness of its complications, and the impact of violent behavior by patients. Most conventional standpoints support that genetic (plexin A2 on chromosome 1q32; Mah et al., 2006) and environmental factors (childhood adversity; Wells et al., 2020) substantially influence the development of SCZ (Yue et al., 2011; Birnbaum et al., 2018). Immune system dysfunction, such as abnormal lymphocyte numbers, and neurotransmitter system dysfunction, such as glutamic receptor dysfunction, have been widely reported as the popular causes (Anticevic et al., 2012; Karpiński et al., 2016). Moreover, neuroimaging studies have provided evidence for abnormal nervous system growth, such as differences in prefrontal cortex activation patterns (Takeda et al., 2017) and default mode network functional connection patterns (Wang et al., 2019) between patients with SCZ and healthy controls. Energy metabolism disorders and mitochondrial dysfunction are also associated with SCZ (Gonçalves et al., 2015). Although research on SCZ has not been lacking in recent decades, its pathological mechanism remains largely unknown, and effective interventions for SCZ patients are still limited.

In recent years, the gut microbiota has been associated with cognitive impairment and mental illness. The critical role of the gut-brain axis in various behaviors and brain disorders has been widely acknowledged (Aziz and Thompson, 1998). The concept of the microbiota-gut-brain axis (MGBA) has been introduced in many studies on interactions between gut microbiota and brain functions. For example, the absence of gut microbiota can influence the brain function of germ-free (GF) animals (Diaz Heijtz et al., 2011; Neufeld et al., 2011), while specific strains of bacterial transplants may change animal behavior (De Palma et al., 2017; Tengeler et al., 2020). The gut microbiota is tightly linked to host brain function *via* neural, endocrine, and immune pathways (Järbrink-Sehgal and Andreasson, 2020); the pathways' communication is usually considered a bidirectional line (Rutsch et al., 2020). Transplantation of fecal microbiota from children with autism spectrum disorders (ASD) into GF mice leads to several ASD-like behaviors, a different microbial community structure, and altered tryptophan and serotonin (Xiao et al., 2021) levels. Similarly, mice receiving fecal microbiota from patients with major depression developed depression-like behaviors and altered gut microbiota metabolism compared to “healthy microbiota” colonization (Zheng et al., 2016).

Although the etiology of SCZ is still unclear, MGBA development provides a new perspective to explain the pathophysiological processes relevant to SCZ. Previous studies have shown that gut microbial disorders can be observed in various rodent schizophrenia models. Using metabotropic glutamate receptor 5 (mGlu5) knockout mice as an SCZ model, Gubert et al. (2020) found that SCZ induced significantly different bacterial diversity and taxonomy. Significant genotype alterations in microbial β-diversity also showed decreased relative richness of the *Erysipelotrichaceae* and *Allobaculum* genera (Gubert et al., 2020). In fecal microbiota transplantation (FMT)-induced SCZ mouse models, lower glutamate, higher glutamine, and GABA levels were observed in the hippocampus in addition to SCZ-related behavior. This was similar to other SCZ mouse models involving glutamatergic hypofunction (Zheng et al., 2019). Meanwhile, fecal samples from patients with SCZ had a decreased microbiome α-diversity index and marked disturbances in gut microbial composition (Zheng et al., 2019). A recent study also showed that 19 gut microbiota taxa, such as phylum *Actinobacteria*, were closely associated with SCZ; the microbial dysbiosis index was positively correlated with gut microbiota-associated epitope diversity and gut IgA levels (Xu et al., 2020).

Although the relationship between gut microbiota and SCZ has been widely accepted, whether the changes in schizophrenia severity are also related to gut microbiota remains limited. It is revealing that the characteristic bacteria highly correlated with symptom changes are still lacking. Therefore, the present study compared the gut microbial communities before and after a short treatment period in SCZ patients. We performed FMT on specific pathogen-free (SPF) mice before or after treatment of SCZ patients to test whether gut microbiota was linked with a decrease in schizophrenic symptoms. In addition, to characterize the gut microbial community assembly, the present study used a neutral community model, random forest, and co-occurrence network to determine the potential characteristic bacteria that may influence the host severity of schizophrenic symptoms.

## Materials and Methods

### Subjects and Fecal Sample Collection

Per the Declaration of Helsinki, this study was approved by the Medical Ethics Committee of the Fourth People's Hospital of Ya' an. All participants provided written informed consent and completed a questionnaire with their general information such as age, sex, race, height, weight, anamnesis, history of smoking, and drinking. The participants were recruited from inpatients receiving antipsychotic treatment and were examined and diagnosed according to the ICD-10 by two trained psychiatric physicians. Clinical psychopathological symptoms were evaluated using the Brief Psychiatric Rating Scale (BPRS; Andersen et al., 1989), Clinical Global Impressions-Severity (CGI-S) Scale, Scale for Assessment of Positive Symptoms (SAPS), and Scale for Assessment of Negative Symptoms (SANS; Malla et al., 1993). An initial stool sample was collected. Then a second stool sample was collected after 14–19 days of treatment (referred to as “before” and “after” treatment). Therapeutic response and side effects were recorded *via* interviews or scales (Clinical Global Impression of Improvement (CGI-I) and Clinical Global Impression of Efficacy (CGI-E). All fresh stool samples obtained from participants were stored at −80°C until DNA extraction.

Supplementary criteria were: (1) age 18–70 years old, male, of Han nationality; (2) absence of any specific drug use for the last 14 days, including antibiotics, probiotics, and prebiotics; (3) absence of any history of gastrointestinal tract disorders or major surgery of the gastrointestinal tract; (4) absence of other psychiatric disorders; (5) completed two-scale assessments before and after treatment (including CGI, BPRS, SAPS, and SANS). After exclusions, 25 of 46 patients met the inclusion criteria for participation. The results of the CGI-I and CGI-E showed that 25 patients showed some improvement after 14–19 days of treatment.

### 16S rRNA Gene Sequencing and Analysis

A total of 200 μl (final elution volume) of fecal DNA samples were extracted using the E. Z. N. A. TM fecal DNA extraction kit (OMEGA Bio-Tek, Norcross, GA, USA). The extracted DNAs were sent to Shanghai Personal Biotechnology Co., Ltd. (Shanghai, China) after measuring DNA quality and integrity by agarose gel electrophoresis and NanoDrop NC2000 spectrophotometer (Thermo Fisher Scientific, Waltham, MA, USA). The specific barcode was combined into the amplification primers, and pair-end 2 × 250 bp sequencing of the bacterial 16S rRNA gene was measured using the Illumina MiSeq sequencing platform and V3–V4 region fragment. The primer sequences used were 338F (5′-ACTCCTACGGGAGGCAGCA-3′) and 806R (5′-GGACTACHVGGGTWTCTAAT-3′). PCR components contained Buffer (5×, 5 μl), Fast pfu DNA Polymerase (5 U/μl, 0.25 μl), dNTPs (2.5 mM, 2 μl), Forward and Reverse primers (10 uM, 1 μl), DNA Template (1 μl) and ddH_2_O (14.75 μl). The amplification procedure consisted of initial denaturation at 98°C for 5 min, degeneration at 98°C for 30 s with 25 cycles, annealing at 53°C for 30 s, extension at 72°C for 45 s, and a final extension at 72°C for 5 min. PCR amplicons were purified and quantified using Vazyme VAHTSTM DNA Clean Beads (Vazyme, Nanjing, China) and a Quant-iT PicoGreen dsDNA Assay Kit (Invitrogen, Carlsbad, CA, USA), respectively.

According to the official tutorial (https://docs.qiime2.org/2020.11/tutorials/), QIIME2 (Bolyen et al., 2018) was used to perform bioinformatics analysis of the sequencing data. The demux plugin, cutadapt plugin (Martin, 2011), and DADA2 plugin (Callahan et al., 2016) were used to demux sequencing and cut primers from the original data, and carry out quality filtering, denoising, merging, as well as the removal of sequences. Non-single-case amplicon sequence variants (ASVs) were aligned to construct a phylogeny between ASVs and fasttree2 (Price et al., 2009). Finally, the species in the abundance table were annotated using the feature-classifier plugin (Price et al., 2009) based on the Navier Bayes classifier and Greengenes 13_8 database.

#### Alpha Diversity, Beta Diversity, and Microbial Composition Analysis

The even abundance was analyzed with the Wilcoxon sum test for alpha diversity (richness and Shannon diversity) using the *vegan* package (https://CRAN.R.project.org/package=vegan). For in-depth analyses, we selected samples in which at least 20% of the ASVs were present and where the sum of the relative abundances exceeded 2.5% (Ju et al., 2014). Filtering removed species that lacked representation largely suppressed non-zero relationships among low-abundance species, reduced network complexity, and facilitated the identification of communities and core ASVs (Ju et al., 2014). Subsequently, we normalized the filtered ASV sequence counts using the TMM method in the *edgeR* package (Robinson et al., 2010). Based on Bray–Curtis's dissimilarities, principal coordinate analysis (PCoA) was used to visualize the structure of the intestinal microbial communities utilizing the package *Phyloseq* (McMurdie and Holmes, 2013). Permutational multivariate analysis of variance (PERMANOVA) was used to evaluate differences in microbial communities between groups with *adonis* in the *vegan* package. The high-abundance microbiota at the phylum and genus levels were clustered to reveal the taxonomic composition changes of the gut before and after treatment. The Wilcoxon rank-sum test was used to compare specific microbial communities between groups. The above analysis was mainly carried out using the R-studio software (V3.1.2).

#### Neutral Community Model Analysis

We needed to assess the potential importance of the stochastic process to the intestinal microflora assembly before and after treatment. As such, this study used a neutral community model (Sloan et al., 2006) to predict the relationship between the frequency and relative abundance of ASVs in the sub-communities after treatment. This could determine the stochastic/deterministic interactions during the succession of the original and disturbing ecosystems (Elzhov et al., 2015). Östman's method was used to calculate the *R*^2^, representing the model's goodness of fit. The assembly of the intestinal microbial community conforms to a random process when *R*^2^ is close to 1; otherwise, it conforms to a deterministic process (Östman et al., 2010).

*Spaa* package (Edgar, 2013) was used to further calculate the Levins niche width index of intestinal microorganisms before and after treatment. A random rearrangement of the *EcolUtils* package simulated the species occurrence frequency. The species whose niche width index exceeded the upper fell below the lower and in the 95% confidence interval as generalist species, specialist species, and neutral taxa, respectively (Wu et al., 2018).

Finally, the empirical C-score was calculated based on the sequential swap randomization algorithm of the *EcoSimR* package (R Core Team, 2018). The zero distribution of the C-score (Stone and Roberts, 1990) was simulated by reassigning the species co-occurrence of the randomization algorithm. The degree of deviation between the empirical C-score and zero distribution of the C-score was determined to evaluate the standardized effect size (SES) for different microbial communities before and after treatment (Gotelli and Mccabe, 2002; Crump et al., 2009). Generally, positive SES values represent the separated co-occurrence pattern of species. Negative SES values represent the aggregated co-occurrence pattern of species. There is no significant difference between the empirical C-score and null distribution, representing the random co-occurrence pattern of species. In other words, the absolute value of SES is considered to be the degree of influence of deterministic processes on microbial assembly (Swenson, 2014). The calculation was based on 1,000 random permutations simulating a zero-distribution C-score to evaluate the co-occurrence patterns of community species before and after treatment.

#### Identifying the Key Species by Random Forest

A combination of random forest (RF) algorithms was used to identify the most influential key pathogenic species. It also helped explain the nonlinear relationship and dependence between microbial community characteristics (Zeller et al., 2014). The RF model introduced the standardized abundance of ASV in all major gut microorganisms. Each was assigned two different important scores (mean decrease accuracy, MDA; mean decrease Gini, MDG) and ranked in descending order to obtain the top 20 ASVs.

#### Co-Occurrence Network and Netshift Analysis

Significant (*p* < 0.05) ASV associations from one sickness state to the other were identified by indicator species analysis to determine the key species that play essential roles in intestinal communities during hospital treatment. These were visualized by the Fruchterman Reingold layout with 10^4^ permutations using the *igraph* package. Meanwhile, *Sparcc* correlation (Friedman and Alm, 2012) was calculated among ASV interactions. The significant interactions (ρ > 0.3 and *p* < 0.05) were retained, followed by the *greedy optimization of modularity* algorithm (Clauset et al., 2004) to identify and visualize the microbial community modules in the co-occurrence network. The co-occurrence network was visualized using the Fruchterman Reingold layout and 10^4^ permutations using the R package *igraph*. Netshift analysis (Kuntal et al., 2019) was used to quantify the community change in the microbial co-occurrence network before and after treatment. Neighbor shift (NESH) scores and Delta betweenness (DelBet) coefficients were used to identify the core microbes that may be considered a “driving force.” In addition, the distance matrix of non-metric multidimensional scaling (NMDS) was sorted directly using the *vegan* package. Then the samples (all) and ASVs with DelBet > 0 in Netshift analysis were projected onto the ordination diagram.

#### Generalized Linear Models

The generalized linear model was conducted using the *mvabund* package. It was used to model the relationship between age, height, weight, education, and six living habits as independent variables, and the abundance of core species as response variables, to analyze the effect of each factor on SCZ development. We used negative binomial regression to make the results more robust and screened out the significant factors for the core pathogenic species based on the significance of 9,999 bootstrap estimates (Benesh and Kalbe, 2016).

### Animals and Fecal Microbiota Transplantation

All experiments were conducted with male C57BL/6J mice, which were maintained in a standard SPF environment (12-h light/dark cycle) at temperature (25 ± 2.0°C) and humidity (55 ± 10%), allowed access to food and water freely. According to the core species, 10 subjects were excluded when the fecal samples were subjected to FMT: (1) neither ASV57 nor ASV86 existed in the samples before treatment; and (2) beneficial bacteria (ASV86) decreased or harmful bacteria (ASV57) increased in the samples after treatment. Fecal samples were collected from the rest of 15 SCZ patients for microbiota transplantation, as described in previous studies (Sun et al., 2018; Liang et al., 2019). One spoon of stool (~1 g) obtained from each patient's fecal sample was suspended and diluted with sterile PBS. The fecal liquid was centrifuged at 800 g for 5 min at 4°C to obtain total bacteria. It was then filtered twice in PBS. About 600 μl of Supernatant fluid was mixed with an equal volume of 40% (volume rate) glycerin-PBS liquid, then diluted. The microbe-containing samples were stored in 20% glycerin-PBS liquid at −80°C until transplantation. The mice were treated with antibiotics to deplete the intestinal flora starting 1 week after habituation by oral gavage once a day for 10 days. After the final gavage of antibiotics for 48 h, mice were randomly divided into two groups. They received oral gavage of the microbiota suspension from patients with SCZ before or after treatment (10 μl/g body weight) for 2 weeks to reconstruct the gut microbiota (hereafter referred to as BT and AT mice). All animal experimental procedures were approved by the Institutional Animal Care and Use Committee of Sichuan Agricultural University.

### Behavioral Testing

Behavioral tests were performed using 12 mice per group to ensure the effectiveness of gut microbiota manipulation. All behavioral tests were conducted 48 h after fecal transplantation and were recorded using a video-computerized tracking system (SMART 3.0; Panlab SL, Barcelona, Spain) or evaluated separately by manual observation by two experienced observers. The investigators were blinded to the treatment conditions of the mice during the experiments and outcome assessment. Animals were given 1 h of habituation in the behavioral testing room before testing began to minimize novelty or stress effects. Behavioral tests were performed in the following order: open field test (OFT), elevated plus maze test (EPMT), three-chamber social test (TCST), novel object recognition test (NORT), and forced swimming test (FST). All tests were performed as previously described with minor modifications (Ikeda et al., 2020; Wang et al., 2020; Zhu et al., 2020b). Tests were performed from the least to the most invasive, with a minimum 48 h interval between tests to minimize the influence of previous test history. All objects used for the tests were cleaned with 70% ethanol between each trial, and the feces and urine in the box were removed.

#### Open-Field Test

Mice were placed individually in an unfamiliar open-field arena (40 × 40 × 45 cm) and allowed to explore freely for 10 min. The video-computerized tracking system analyzed the total distance traveled (considered an evaluation of locomotor activity) and the time spent in the central zone (15 × 15 cm squared arena). Changes in distance and time in the central zone indicated different anxiety levels (Ikeda et al., 2020).

#### Elevated Plus-Maze Test

The plus-shaped maze consisted of two open and two closed arms (25 × 5 cm, 20 cm walls, 50 cm above the ground), with arms of the same type facing opposite each other. Mice were placed in the central square (5 × 5 cm) of the maze, facing one of the closed arms. The time spent in the closed and open arms was manually recorded using filmed video during an observation period of 5 min. The ratio of time spent in the open arms was considered a measure of anxiety (Zhu et al., 2020b).

#### Three-Chamber Sociability Test

The test setup was a box containing three chambers (60 × 40 × 22 cm) separated by Plexiglas walls with openings inside to allow access to each chamber. In the habituation trial, test mice were individually placed in the center chamber and allowed to freely explore all three chambers for 10 min. Subsequently, a small, round wire cage with unfamiliar sex- and strain-matched conspecific (as a stranger) was placed in one of the side chambers, and the same empty cage was placed in another side chamber. In the sociability trial, test mice were placed in the center chamber and allowed to freely explore all three chambers for 10 min. At the end of the 10-min sociability trial, a novel object was placed in an empty cage. The test mice were free to explore the mouse from the previous sociability test (stranger 1) and the novel mouse (stranger 2) for 10 min. The amount of time spent in each cage was recorded using a video camera (Zhu et al., 2020b).

#### Novel Object Recognition Test

Mice were placed in a 40 × 40 × 45 cm box alone to explore freely for 30 min for habituation under dimly-lit conditions. Two similar objects (smooth pebbles of different colors) were placed in the center of the two halves of the box, and the mice were allowed to explore for 10 min before returning to their home cage. After 24 h, one of the objects was replaced by a tube cap. The mice were placed in the box again for the test session and allowed to explore the familiar and novel objects for 5 min. The discrimination index (DI), a measure of memory function, was defined as the ratio of time spent exploring the novel object to the total time spent exploring both objects during the test session (Wang et al., 2020).

#### Forced Swim Test

Mice were individually placed in an open, transparent plastic cylinder (diameter: 20 cm; height: 40 cm), which contained 20 cm of water, kept at 24°C ± 1°C. The duration of the mice's immobile behavior was manually recorded in the FST, lasting for 5 min (Zhu et al., 2020b).

### Statistical Analysis

All graphs and statistical analyses were performed using the GraphPad Prism software version 8 and IBM SPSS 26.0. Data are expressed as mean ± standard deviation (SD). Differences between the two groups were assessed using a two-tailed Student's *T*-test. Nonparametric tests, including the Kruskal–Wallis test and Wilcoxon rank-sum test, were used to assess the two groups' 16S rRNA gene sequencing data. Statistical significance was set at *p* < 0.05.

## Results

The patients' demographic data and clinical parameters are shown in Supplementay Tables 1, 2. There was a significant difference in the BPRS, SAPS, and SANS scores before and after treatment.

### DNA Sequencing and Screening

**Figure 1:**
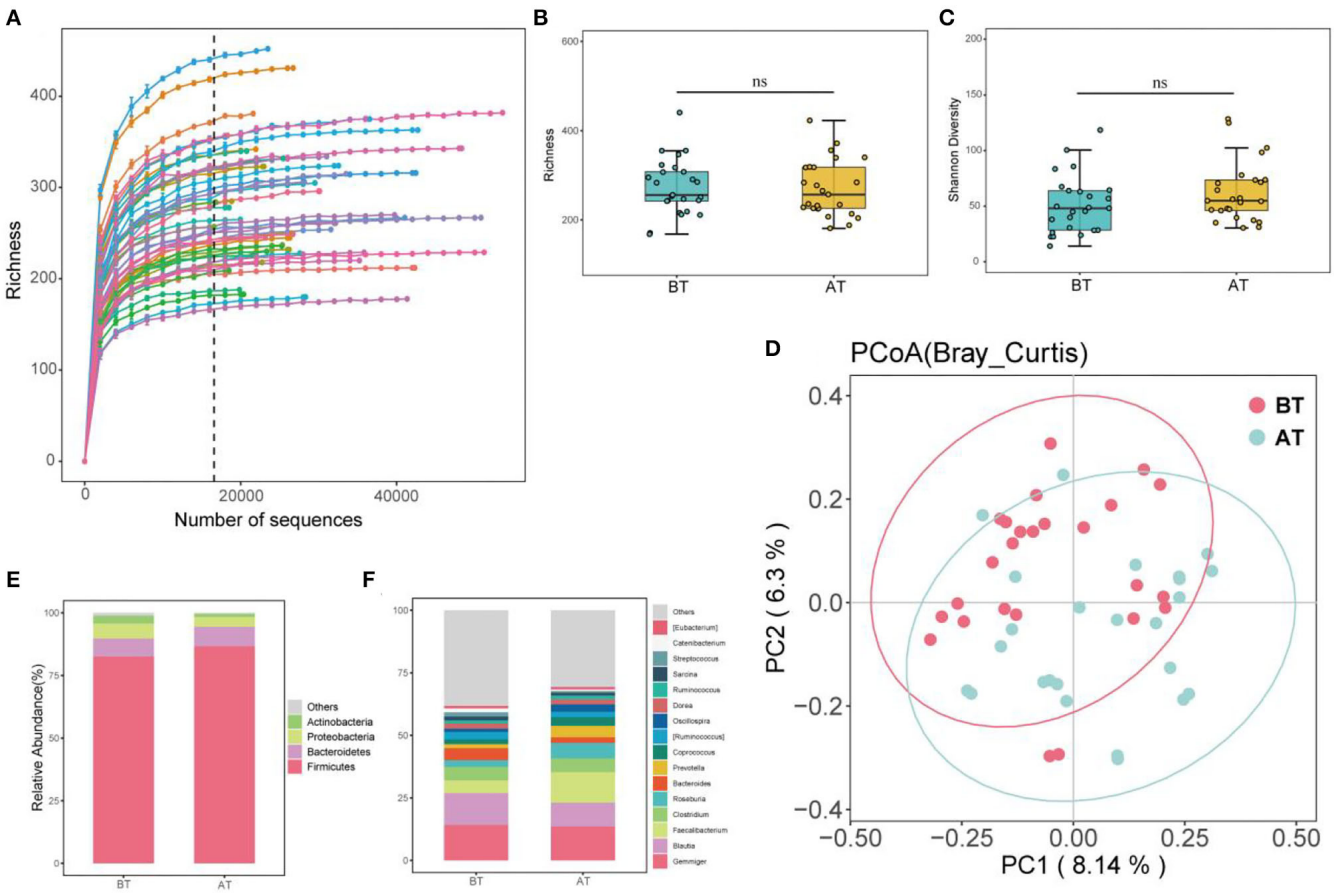
Alpha, Beta diversity, and gut microbial composition. **(A)** Rarefaction curves for intestinal microbiota observed ASV richness. The color lines indicate BT and AT samples, the dashed black line indicates the selected rarefaction depth used to generate the box plots below each curve, 16,589 seqs/sample for microbial communities. **(B)** Community richness of intestinal microbiota in BT and AT samples (Richness); **(C)** Community diversity of intestinal microbiota in BT and AT samples (Shannon diversity), ns, no significant. **(D)** Principal coordinate analysis (PCoA) of intestinal microbiota of mice under different group. **(E)** Relative abundance of dominant phylum taxa (>1%) in intestine from each group. **(F)** Relative abundance of dominant genus taxa (>1%) in intestine from each group.

A total of 2,098,961 clean reads were generated from the MiSeq platform and retained after filtering for low-quality reads. The high-quality reads were 2,9319 per sample. In total, 6,667 features (ASVs) were identified. The rarefaction curves showed that all samples reached the platform level (Figure 1A), indicating that our sequencing depth was suitable for mining the microbial community in the fecal samples.

### Changes in Gut Microbial Alpha Diversity and Beta Diversity Before and After Treatment in SCZ Patients

After treatment in the hospital, the species richness (Figure 1B) and Shannon diversity (Figure 1C) of the microbiota community increased in patients with SCZ. Still, the differences were not significant compared to before treatment (*p* > 0.05). To compare the structural differences of gut microbiota before and after treatment (referred to as BT and AT groups), the distance matrix based on Bray-Curtis and the PCoA was used to evaluate and visualize beta diversity (Figure 1D). Figure 1D shows significant differences between the BT and AT groups, and PERMANOVA further confirmed significant differences between the two groups (*p* < 0.05).

### Altered Microbial Compositions of the Gut Microbiota After Treatment in SCZ Patients

At the phylum level, 18 taxa were observed in all samples, of which four different taxa were identified as highly abundant phyla (relative abundance >1%). *Firmicutes* (84.61%), *Bacteroidetes* (7.48%), and *Proteobacteria* (4.40%) were the first, second, and third most dominant phyla, respectively. Together with *Actinobacteria* (2.85%), they accounted for 99.26% of the total sequence (Figure 1E). *Firmicutes* were the dominant species at the phylum level, with relative abundances of 82.60% and 86.64% before and after treatment, respectively (Figure 1E).

**Figure 2:**
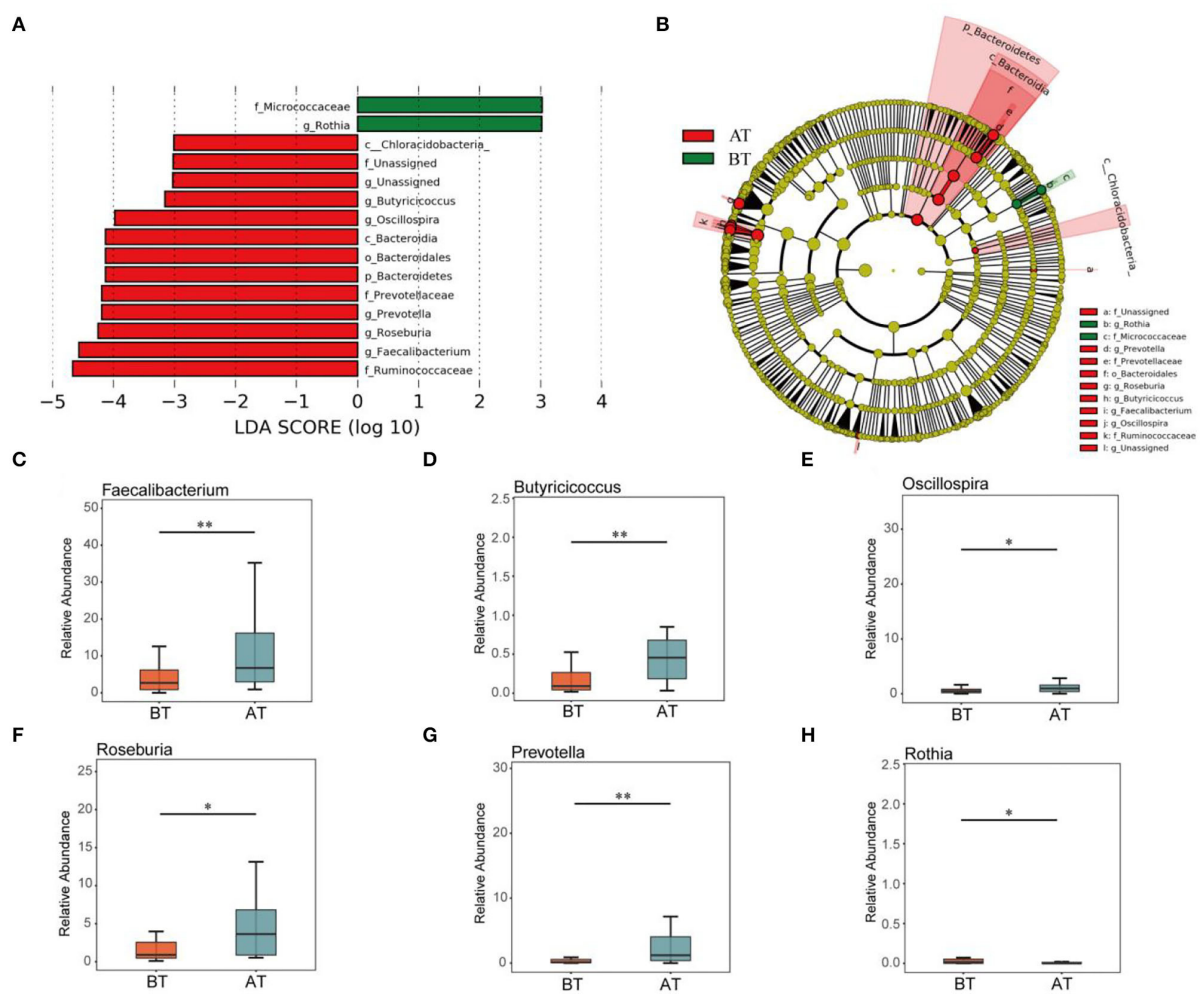
Comparison of intestinal difference groups between BT and AT groups. LEfSe analysis of key taxa of gut microbiota in mice between the BT group and AT group. **(A)** LDA score plot of bacterial taxa with LDA scores higher than 4. **(B)** Taxonomic cladogram derived from LEfSe analysis of 16S rRNA sequences. **(C-H)** Boxplots showing differences in the relative abundance of specific taxa between BT and AT patient. Specific taxa in figure **(C–H)** were *Faecalibacterium* **(C)**, *Butyricicoccus* **(D)**, *Oscillospira* **(E)**, *Roseburia* **(F)**, *Prevotella* **(G)**, and *Rothia* **(H)**. *p < 0.05, **p < 0.01.

At the genus level, 238 taxa were detected in all samples (Figure 1F), of which *Gemminger, Blautia, Facecalibacterium, Clostridium, Roseburia, Bacteroides*, and *Prevotella* accounted for 65.63%. *Gemminger* was the dominant species at the genus level, with relative abundances of 14.17 and 13.57% before and after treatment, respectively. *Blautia* (a relative abundance of 12.77% and 9.56% before and after treatment) and *Facecalibacterium* (a relative abundance of 5.08% and 12.23%, before and after treatment), respectively, were the second and third dominant species at the genus level. Furthermore, LEfSe was used to identify biomarkers before and after the treatment (Figures 2A,B). The biomarkers in the BT group included *Chloracidobacteria, Butyricicoccus, Oscillospira, Bacteroidia, Bacteroidales, Bacteroidetes, Prevotellaceae, Prevotella, Roseburia, Faecalibacterium, Ruminocaoccacceae*. *Micrococcaceae* and *Rothia* were biomarkers of the AT group. According to the Metastats analysis, the relative abundance of *Faecalibacterium, Butyricicoccus, Oscillospira, Prevotella*, and *Roseburia* increased significantly after treatment (*p* < 0.05, or *p* < 0.01, Figures 2C–G). The relative abundance of *Rothia* decreased significantly after treatment (*p* < 0.05, Figure 2H).

### The Assembly Mechanism of Gut Microbiota in Patients Before and After Treatment

**Figure 3:**
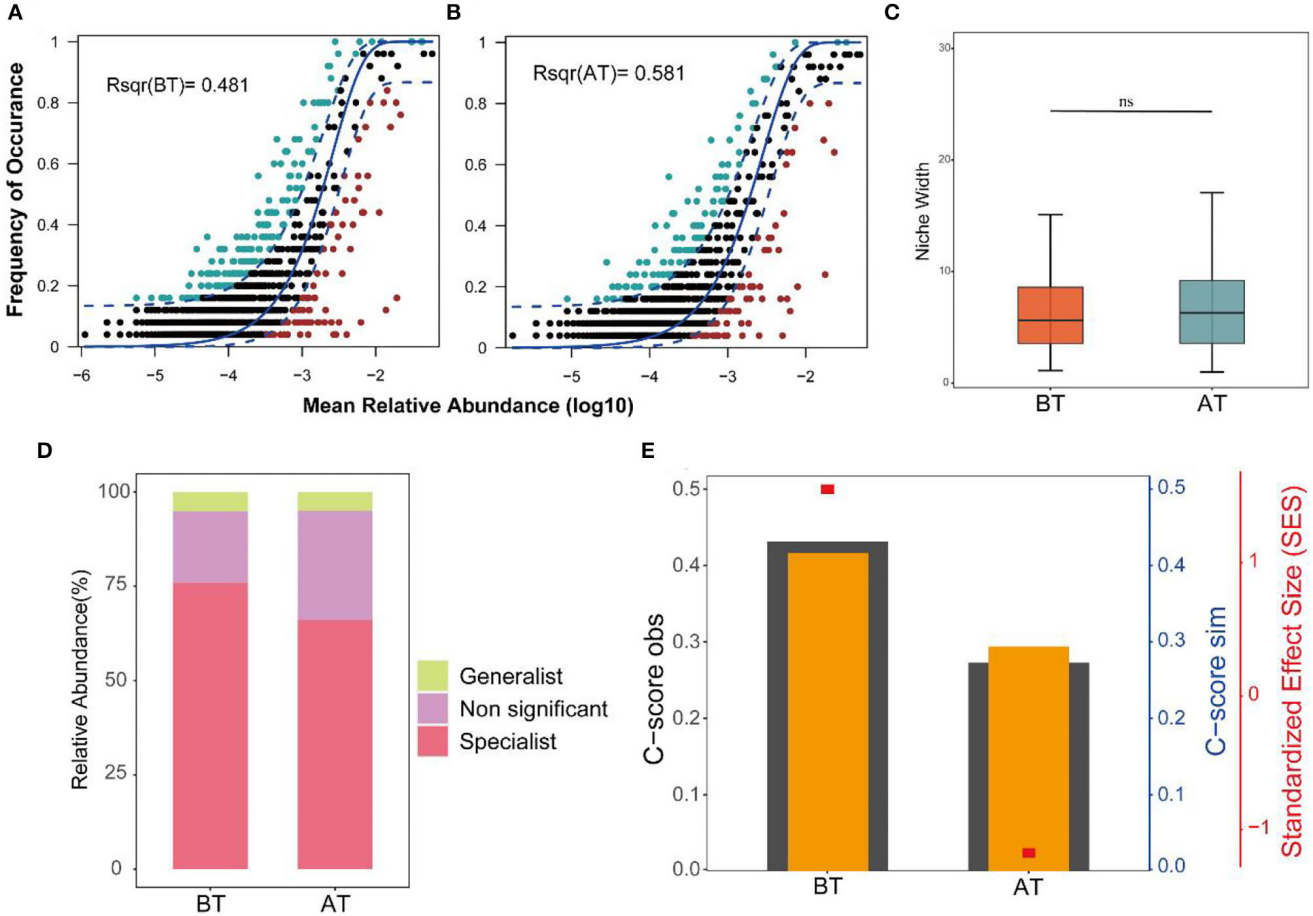
Ecological processes shaping the gut community assembly in BT and AT patients. **(A,B)** Predicted frequency of occurrence of gut microbiota in the BT group **(A)** and AT group **(B)**. The solid blue line is the best fit to the neutral community model (NCM), and the dashed blue line expresses 95% confidence intervals around the NCM prediction. ASVs that occur more or less frequently than predicted by the NCM are shown in lightgreen and lightred, respectively. *R*^2^ indicate the fit to this model. **(C)** Boxplot shows the niche width index distribution of microbial community of gut in BT and AT samples. **(D)** Relative contributions of generalized and specialized species to BT group and AT group of intestinal microbiotas. **(E)** C-score metric using null models. The values of observed C-score (C-score_obs_) > simulated C-score (C-score_sim_) represent non-random co-occurrence patterns. ns, no significant.

The neutral community model adequately estimated most of the relationships between the occurrence frequency of ASVs and their relative abundance changes. The explaining rates for the BT and AT groups were 48.1 and 58.1% of community variation, respectively. This indicates that the relative contribution rate of the random process increased significantly after treatment (Figures 3A,B). In contrast, the Levins niche width indices of intestinal microorganisms changed indistinctively before and after treatment (Figure 3C). Both generalist and specialist species were widespread in the gut microbes of the BT and AT groups. Still, the proportion of specialist species in the gut microbes of the patients was lower after treatment than before treatment (Figure 3D). More importantly, the C-score results showed that AT's SES decreased, indicating an increase in the importance of random processes for the gut flora (Figure 3E).

### Random Forest and Co-Occurrence Network Identified the Key Gut Species

**Figure 4:**
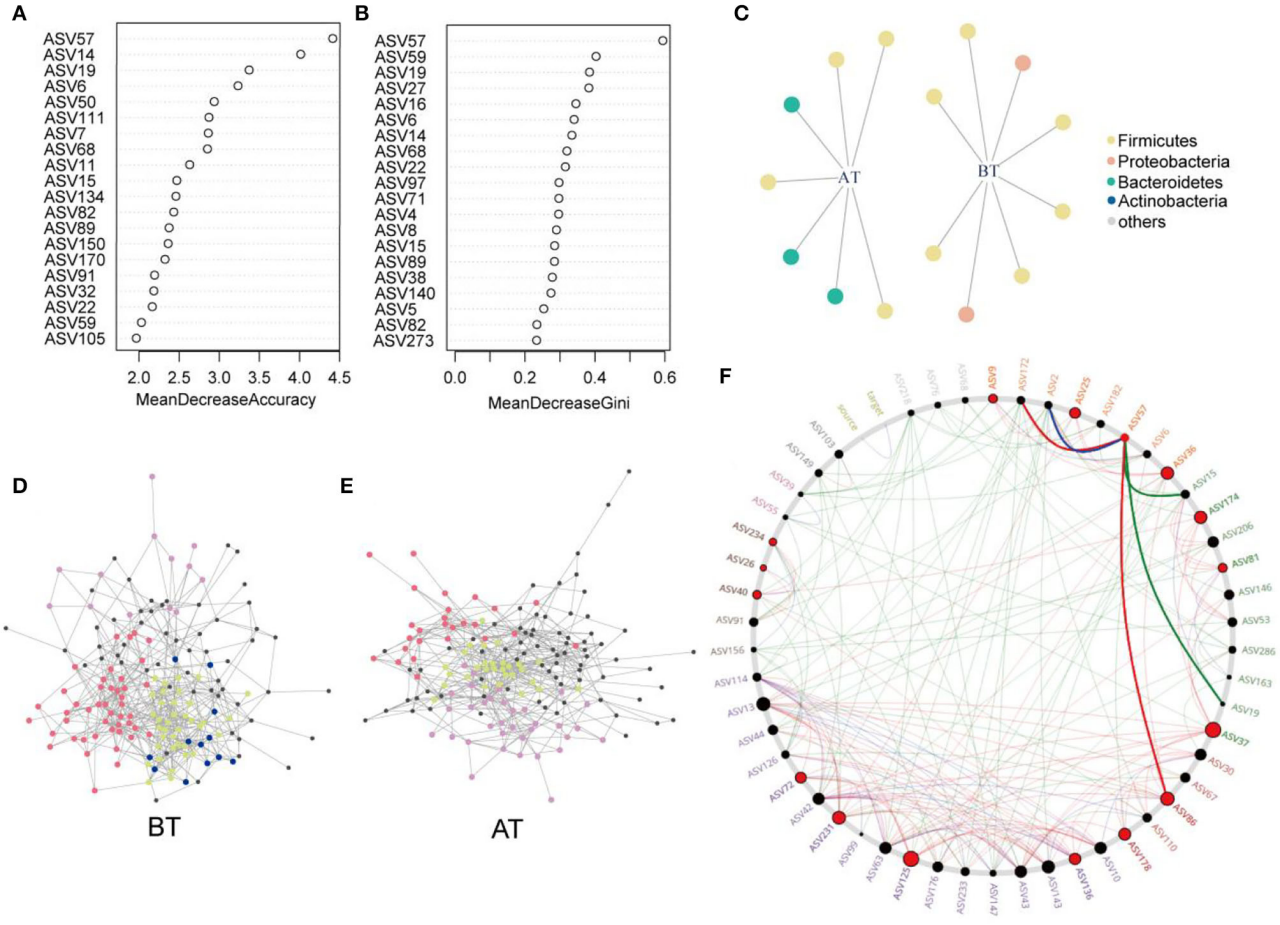
Identification of core ASVs and microbial network analysis. **(A,B)** Random forest showing the top 20 features of the intestinal microenvironment distinguished in the pre- and post-treatment datasets. Each feature (ASV) was assigned two importance score of mean decrease accuracy **(A)** and mean decrease Gini **(B)**. **(C)** Bipartite network showed ASVs specific to before or after treatment identified using indicator species analysis in gut microbial communities. The circle indicates that single species are positively and significantly correlated (*p* < 0.05) with one or more gut microenvironments (the link given by the line). Different ASVs belonging to same phylum level are given the common color. Co-occurrence sub-networks of **(D)** before treatment or **(E)** after treatment. Circles for microbial ASVs are represented by nodes. And partial nodes are colored by association with different modules, which include indicator species corresponding to different gut microenvironment. **(F)** Common sub-networks analysis of BT group and AT group. All nodes belonging to the same community were randomly assigned similar colors. Gray nodes represented ASVs appearing in both the variable group. Big and red node can be considered as “drive microbiota” Red line indicate only in the AT group of sub-networks of interactions. Green line indicates only in the BT group of sub-networks of interactions. And blue line expresses the interactions in both sub-networks.

**Figure 5:**
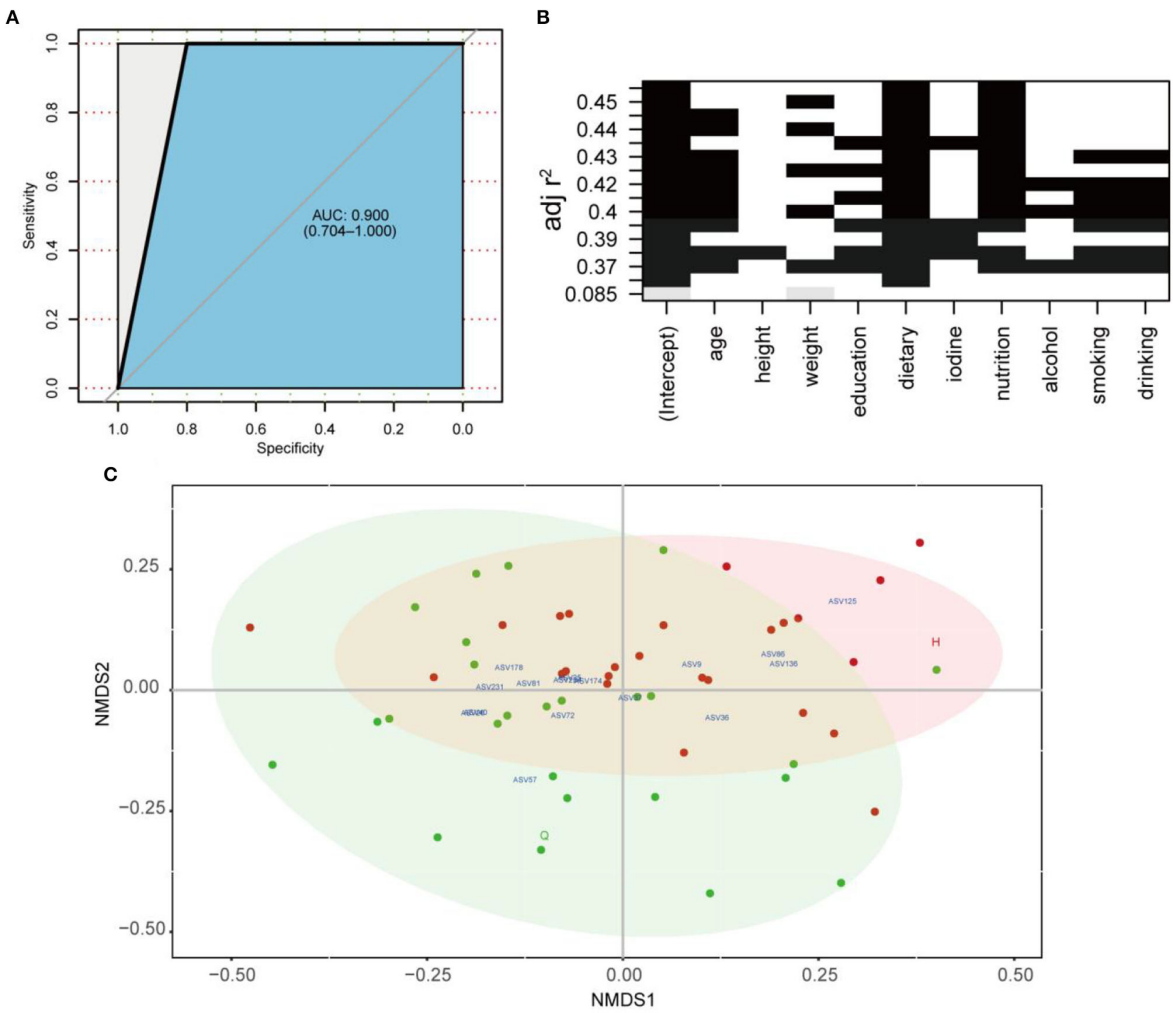
**(A)** ROC according to 40 samples of the testing set calcualted by cross-validated random forest model. Area under ROC (AUC) and the 95% confidence intervals are also shown. **(B)** All-subsets Regression. The horizontal axis represents the predictive variables, and the vertical axis represents the accuracy of the regression (adjusted *R*^2^), which shows how the accuracy of the regression when different combinations of predictive variables are considered. **(C)** Non-metric multidimensional scaling (NMDS) based on the Bray–Curtis distances. Core ASVs (DelBet > 0) are labeled blue and projected as biomarkers to different locations pre- or post-treatment.

Eighty percent of the samples were randomly selected as the training set for RF analysis. The model was constructed using the training set to classify the two states of patients before and after treatment. Meanwhile, the receiver operating characteristic curve (ROC) was measured by testing set as shown in Figure 5A. The area under the curve (AUC) of the testing set reached 0.9, indicating good prediction accuracy on the test dataset. Moreover, the MDA and MDG scores revealed that ASV57 was one of the main features distinguishing the different states before and after treatment (Figures 4A,B). According to the indicator species analysis, there were no shared ASVs between the two groups. This indicates that the characteristic bacteria before and after treatment did not have sufficient similarities in terms of habitat preference. Although the numbers of indicator species in the two groups were similar, the species were completely different (Figure 4C).

Subsequently, the distribution patterns of the indicator species ASV and its community modules in the gut microbiota symbiotic network were found (Figures 4D,E). The observed higher average path length and betweenness centrality of the AT sub-network pointed toward sufficient rewiring compared to the BT sub-network. The lower nodes, connections (edges), average degree, and density in the AT sub-network indicate lower information transport. This might suggest attenuated colonization activity of harmful microbes (Table 1). The microbiome within the module (except for the gray nodes) produced corresponding responses to different states (i.e., BT/AT). Assuming that the colored network nodes were the key regions for further exploring of core species, the key regions of the co-occurrence subnetworks of the AT and BT groups were extracted and subjected to Netshift analysis. The results showed that ASVs with high NESH score were ASV 37, ASV 136, ASV 86, ASV 40, ASV 81, ASV 174, ASV 36, ASV 57, ASV 25, ASV 125, ASV 231; and ASV's with DelBet > 0 were ASV 26, ASV 72, ASV 234, ASV 9, ASV 178 (Table 2). These 16 species appeared as large red nodes in the common subnetworks. They were also identified as “driving microbes” that may alter the connections between microbes–ASV 57, which was previously identified as the most important biomarker for different states in the RF model. The red edge connected to ASV 57 indicated that it interacted only in the AT group sub-network, including ASV 86 and ASV 172. The green edge connected to ASV 57 indicated that it interacted only in the BT sub-network, including ASV 15 and ASV 19. The blue edge connected to ASV 57 indicated that it interacted in two groups of sub-networks, including ASV 2 (Figure 4F). Similar results were observed in the NMDS analysis (Figure 5C), with species scores for ASV 57 predicted across BT samples. These results suggested that ASV 57 may be one of the main causes of schizophrenia development.

**Table 1:** Properties of before treatment and after treatment co-occurrence sub-networks.

**Community**	**Node**	**Edge**	**Ave D**	**Ave PL**	**Density**	**Bet C**
BT	107	436	8.15	2.9	0.77	0.05
AT	89	298	6.70	3.1	0.76	0.13

*Ave D, average degree; Ave PL, average path length; Bet C, betweenness centralization*.

**Table 2:** Properties of network nodes in BT group and AT group.

**S_ID**	**n(BT)**	**n(AT)**	**Core (AT)**	**Exclusive**	**Jaccard-score**	**NESH-score**	**DelBet**
ASV 37	1	11	6	11	0.000	2.650	1.000
ASV 136	4	13	7	9	0.308	1.985	0.723
ASV 86	2	10	5	9	0.091	2.327	0.523
ASV 40	4	4	2	3	0.143	1.486	0.448
ASV 81	3	6	3	4	0.286	1.552	0.441
ASV174	1	5	3	5	0.000	2.167	0.320
ASV 36	3	7	4	7	0.000	2.167	0.316
ASV 57	3	3	2	2	0.200	1.333	0.274
ASV 25	1	5	3	4	0.200	1.867	0.156
ASV 125	1	11	7	11	0.000	2.650	0.144
ASV 231	5	9	6	9	0.000	2.243	0.088
ASV 26	1	2	2	1	0.500	1.067	0.077
ASV 72	2	6	5	5	0.143	1.905	0.064
ASV 234	4	3	2	2	0.167	1.300	0.063
ASV 9	3	4	3	3	0.167	1.533	0.011
ASV 178	1	4	4	4	0.000	2.067	0.003

*n(BT/AT), node degree of co-occurrence in the BT/AT group; Core (AT), core degree of nodes in AT group network. Exclusive indicates the new changes of the node in the AT group. Jaccard-score and NESH-Score represent the core degree of nodes in the network. DelBet indicates the betweenness of the node*.

### The Relationship Between Clinical Parameters and the Key Species

To improve the model accuracy, first, the optimal model for the independent variables in the different subsets was evaluated comprehensively through all-subset regressions (Figure 5B). According to the principle of seeking a parsimony model and low collinearity between variables, flavors, and intake of fruits and vegetables were selected from 10 independent variables. Then a negative binomial model was constructed between them and ASV57 abundance. The whole model was significant, and fruit and vegetable intake and dietary flavors substantially affected the proliferation of ASV 57 (*p* < 0.01). In other words, ASV 57 abundance was associated with fruit and vegetable intake and flavors, regardless of other factors.

### Transplantation of SCZ Patient Microbiota Before and After Treatment Induces Different Behaviors in SPF Recipient Mice

**Figure 6:**
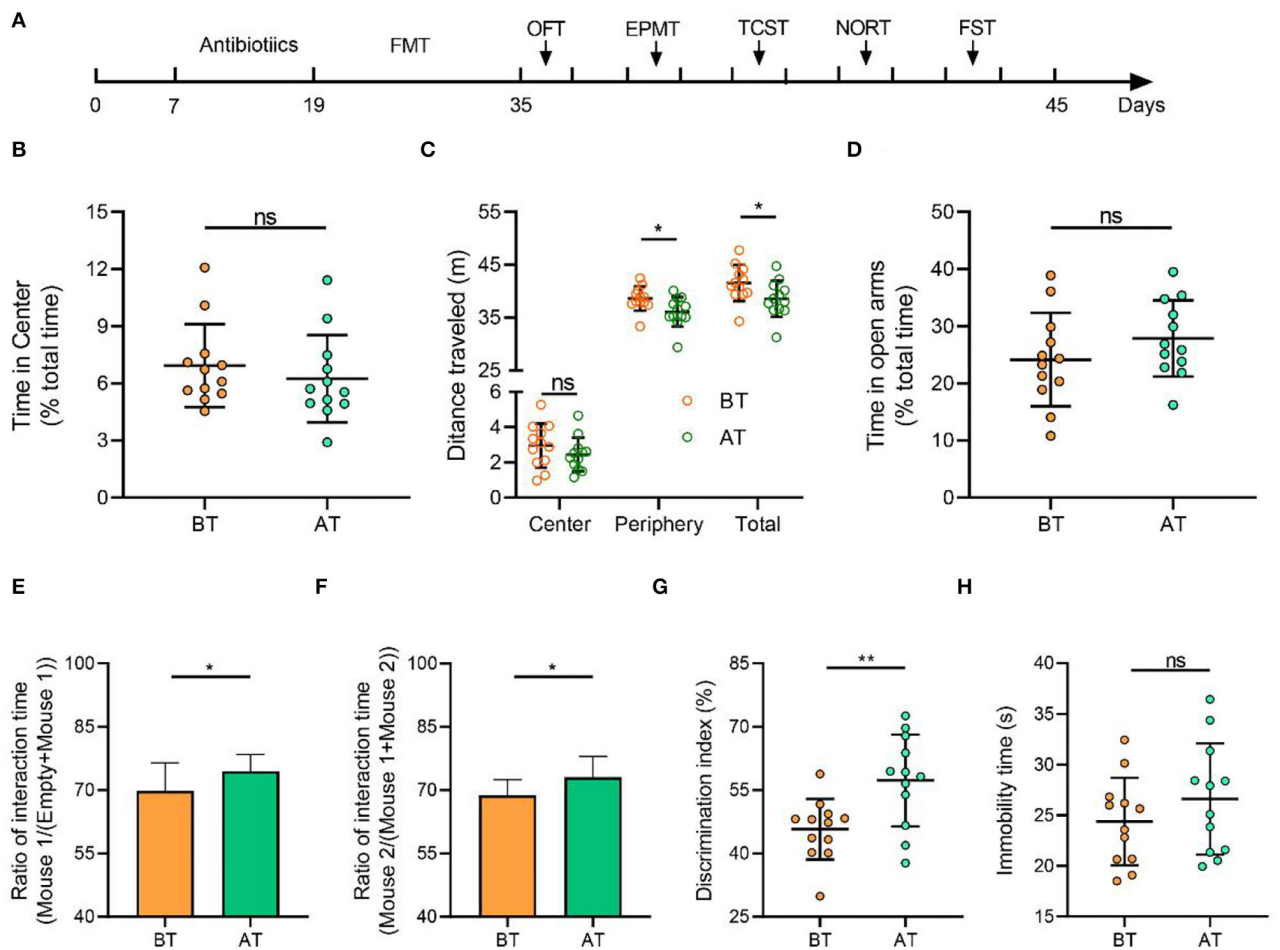
Gut microbiota from patients with SCZ (after treatment) induced a lower level of hyperkinetic behavior and improved sociability, social novelty, and memory in mice than before treatment but had no effects on anxious state and depressive behavior. **(A)** Schematic diagram of mouse treatment and behavioral testing. Mice were given a cocktail of antibiotics to eliminate gut microbiota and received oral gavage of the microbiota suspension from before or after treatment patients with SCZ. A series of behavioral tests were carried out 48 h after the last FMT. OFT open field test, EPMT elevated plus maze test, TCST three-chamber social test, NORT novel object recognition test, FST forced swimming test. **(B)** In the 10-min open field test, both groups of mice spent similar amounts of time in the central arena. **(C)** The cumulative distance (meters) traveled in the different zones, the representative traces of mouse activity. **(D)** Time in open arms of the elevated plus maze during 5-min exploration did not vary between two groups. **(E)** AT mice had obvious changes in sociability compared with BT mice. **(F)** AT mice had obvious changes in social novelty compared with BT mice. **(G)** Novel object recognition test. AT mice spent significantly more time exploring the novel object compared with BT mice, indicating improvement of impaired recognition memory. **(H)** Immobility in the forced swimming test (FST, *p* = 0.138) was similar between BT and AT mice. Means ± SD were presented in **(B–H)** and each circle represents individual mouse (*n* = 12 per group). *P*-values were determined by the Independent Student *T*-test. ns, no significant. *p < 0.05, **p < 0.01.

A multitude of behavioral tests were performed within 10 days of the FMT on the BT and AT mice (Figure 6A). Although there was no difference in the timing at the central area over the 10 min OFT (Figure 6B), AT mice showed decreased distance traveled (Figure 6C) compared to the BT mice. This indicates lower levels of hyperkinetic behavior. In the TCST, an untreated SPF mouse was used as a stranger. The mice's sociability and preference for social novelty were quantified based on the ratio of the time spent around the wire cage with a stranger mouse vs. the total time spent around the empty wire cage. In the sociability test, AT mice showed a significantly higher ratio of interaction time (around the wire cage with the stranger mouse) than BT mice (Figure 6E), indicating an improvement in sociability. In the social novelty preference test, AT mice showed a significantly higher ratio of interaction time (around the wire cage with the novel stranger mouse) than BT mice (Figure 6F). This indicates an improved preference for social novelty. The discrimination index of NORT showed that AT mice had improved memory impairment for previously explored objects (Figure 6G). In the EPMT, no significant difference was detected in the time spent in the open areas between the BT and AT mice (Figure 6D). During the FST, a similar result was observed for the immobility duration (Figure 6H).

## Discussion

We used high-throughput 16S rRNA sequencing of fecal samples to conduct microbiome profiling and identified the core species related to SCZ severity. While previous studies have found other evidence of intestinal microbial shift using 16S rRNA sequencing in patients with SCZ, this is the first study to explore the intrinsic changes and core microbes in the gut microenvironment at different baseline phases before and after treatment in the same patients. Although the diversity and composition of the gut microbiome vary greatly among different patients in the same phase, we were able to detect representative community characteristics and variation patterns in almost all samples. Specifically, we have been attempting to analyze the microbial compositions and community assembly before and after treatment in-depth to gain insights into the influence of core species on each of these phases in patients. The combination of treatments is thought to be the greatest source of change in the gut microbiome when other conditions are controlled. Overall, the pattern of separation of the different groups of microbial structures from baseline to post-treatment was demonstrated by statistical tests using PERMANOVA. Conversely, intestinal community richness and diversity within patients (α-diversity) were not significantly different between the baseline and after treatment. In other studies (Li et al., 2020; Nguyen et al., 2021), we did not observe strong evidence for an obvious difference in the amount or distribution (α-diversity) of the microbiome in patients with schizophrenia compared with controls or after treatment. Those results are consistent with this study. In general, higher α-diversity is regarded as the main characteristic of improved intestinal health. It is widely accepted that greater microbial richness and diversity increase intestinal ecosystem resistance and adaptability due to increased functional redundancy at the metabolic level and a greater level of defense against pathogen invasion (Lozupone et al., 2012; Larsen and Claassen, 2018). Nevertheless, gut microbiota research in humans is increasingly finding that α-diversity data are of limited utility as a reference for intestinal health or for discriminating between different treatments. This has been proven in the intestinal microbiome in other neurological and psychiatric conditions related to autism spectrum disorder (Ho et al., 2020), multiple sclerosis (Mirza et al., 2020), and Parkinson's disease (Nuzum et al., 2020). In addition, the Bray–Curtis distance showed that individual patients after treatment harbored a clearly varied microbial community compared to the composition structure observed before treatment. At the same time, overall distributions had greater variability across the PCoA space in the two groups. According to Nguyen et al. (2021), there was no difference in any alpha diversity of intestinal microbiota between schizophrenias and controls, while significant inter-group differences were observed in unweighted Unifrac and Bray–Curtis distances. Interestingly, similar results were demonstrated for functional analyses in their study. It is well-known that β-diversity distances can display whether variations exist between different treatments. In contrast, they cannot explain what the variations are or at what level of species they occur.

Next, we focused primarily on the microbial composition and comparative analysis of different taxa in the gut. In this study, the intestinal bacteria of patients with schizophrenia mainly consisted of four dominant taxa: *Firmicutes, Bacteroidetes, Proteobacteria*, and *Actinobacteria*, which accounted for more than 90% of the total microbial count in the gut. A similar conclusion was demonstrated in the previous studies (Shen et al., 2018). Meanwhile, the abundance of *Faecalibacterium, Butyricicoccus, Oscillospira, Roseburia*, and *Prevotella* was much higher in the gut of AT patients than in BT patients. In contrast, most other taxa were found to be biomarkers in the AT group, such as *Rothia*. *Faecalibacterium*, an anti-inflammatory butyric-producing taxonomy, has been shown as a common bacterial signature in psychiatric disorders, including major depression, anxiety, and schizophrenia (Nikolova et al., 2021). Moreover, lower *Roseburia, Butyricicoccus, Oscillospira*, and *Prevotella* abundances have also been observed in psychological and behavioral abnormalities (Geirnaert et al., 2015; Shen et al., 2018; Michels et al., 2019; Yang et al., 2021). In this study, the taxa abundance of the above-mentioned microbiota appeared to increase considerably under hospital treatment. It is worth noting that *Oscillospira* and *Butyricicoccus* have been widely considered next-generation probiotic candidates and therefore have great potential for development and application in food, health care, and biopharmaceutical products (Geirnaert et al., 2015; Yang et al., 2021). Finally, gut *Rothia* has been confirmed to be involved in the immune-inflammatory response of the body and is related to the development of allergic diseases and alcoholic fatty liver disease (Simonyte Sjödin et al., 2016; Sverrild et al., 2017; Maccioni et al., 2020).

Unfortunately, the mechanisms of these divergences in gut microbiota composition, the clinical or treatment significance of these changes, and the patterns of psychological disorders that these processes may reveal in patients remain unclear. Therefore, further research will combine more in-depth approaches, including assembly mechanisms of microbial communities (to identify the underlying causes of the gut microbial community), RF disease classifiers (to evaluate diagnostic values of gut microbes), and microbial interaction network analysis (to identify valuable pathogenic bacteria and potential probiotics). The above results may provide greater insight into the development of schizophrenia and assist in understanding how relevant intestinal core species are related to pathological processes that potentially affect mental health.

Regarding the assembly of gut microbial communities, our study demonstrated that treatment in hospitals has an important effect—it interferes with the balance between stochastic and deterministic processes. The variation in the gut microbial community explained by stochastic processes increased from 48.1% before treatment to 58.1% after treatment. However, the niche breadths of the microbial communities in the AT group did not show significant changes compared to those in the BT group, demonstrating that the microbial community assembly was more strongly influenced by stochastic processes in the AT groups. Deterministic processes are likely to influence habitat specialists with a narrower niche breadth than generalists with a wider niche breadth (Pandit et al., 2009; Wu et al., 2018). In addition, C-score analysis indicated that the SES decreased with hospital treatment. The above findings demonstrated that stochastic processes greatly influenced the gut microbial community assembly with treatment in the hospital. This was likely because treatment processes presented a novel but stable random equilibrium between the loss and gain of taxa in the gut microbial community.

Subsequently, we constructed a prediction model of 164 microbial ASVs to explore specific gut microbes associated with schizophrenia and estimate their diagnostic values. This model indicated high accuracy (AUC = 0.9) and was validated in the testing set, showing that an effective species-based classifier model was not significantly influenced by clinical indicators such as age, height, weight, and gender. Among the 164 ASVs included in the classifier, ASV 57 was significantly enriched in schizophrenia and was annotated as *Ruminococcus*. Previous studies have shown that *Ruminococcus* spp. is significantly associated with Crohn's disease, IBD, and type 2 diabetes (Hall et al., 2017; Baumgartner et al., 2021; Ruuskanen et al., 2022).

Indicator species analysis and Netshift analysis were used to explore the response pattern of the intestinal microbial community for treatment in the hospital and to identify core species. Indicator species analysis is more practical for determining the habitat preferences of species (De Cáceres et al., 2010). Furthermore, we identified key sub-communities (modules) in the co-occurrence network based on the indicator species ASVs. Modularity refers to the natural partitioning of nodes into different groups or modules within a network. Each module is ecologically considered a collection of closely related species and functional units. These species may have ecologically similar functions and can be grouped into the same modules (Baldassano and Bassett, 2016). In the co-occurrence network, we detected four and three microbial community modules in the BT and AT groups, respectively, constituting a common subnetwork under different states. In the present study, we tried to perform an analysis called “Netshift” to understand and identify the “drive microbes” from before and after treatment microbiome datasets from the basis of each subnetwork. It is necessary to consider the abundance of microbes and quantify the variations among the microbial associations to make an intestinal microbiome-based inference. We focused on alterations in the respective associations of nodes between different networks during the reconnection of microbial networks. A node (microbial species) has a similar degree of post-treatment and pre-treatment (total connections to other resident taxa). Still, it might have a completely different set of associations (members participating in the connections). Thus, a centrality index, such as the node degree, is usually used to quantify these changes, but it cannot accurately evaluate associations. In addition, a Jaccard index can be used to quantify the degree of change in the interaction partner of each node between the two networks. However, its results were non-directional. The “Neighbor shift (NESH) score” quantifies specifically enriched interaction partners after treatment, resulting in directional changes of individual node associations. An increase in the medial of a treated gut microbe was used as an index to quantify the increased importance of the considered nodes. A species with a set of altered associations after treatment (determined by high NESH values), but still increasingly important for the entire network (determined by positive DelBet), must be critical in microbial interactions and is predicted to be the “drive microbes” (Kuntal et al., 2019). An increase in delta betweenness (DelBet) of an ASV in the AT group is considered a characteristic to quantify the increase in the importance of this ASV. A gut species in AT patients with a changed set of correlations (high NESH value), while still being increasingly important (positive DelBet) for the microbial network, necessarily holds core importance in microbial interplay and is identified as a key species (Kuntal et al., 2019). According to our results, ASV 57 was also detected as one of the “driver species.” ASV57 proved to have negative roles and therefore can be identified as a harmful driver related to schizophrenia. In the common sub-network, the AT patient-specific correlations (displayed as red edges) of ASV 57 pointed toward other core species such as ASV 86, namely *Oscillospira*, which had positive DelBet and high NESH scores and were therefore inferred to have roles in resisting pathogen colonization. *Oscillospira* extensively colonizes the intestinal tracts of animals and humans. Many studies have shown that the gut *Oscillospira* is associated with Parkinson's disease, autism, and colitis disease (Zhai et al., 2019; Liu et al., 2020; Vascellari et al., 2020; Zhang et al., 2020). *Oscillospira* is closely associated with the central nervous system and degenerative diseases. According to a previous study, patients with Parkinson's disease also have a high abundance of *Oscillospira* (Zhang et al., 2020). However, in another study, Zhai et al. (2019) identified the opposite trend by sequencing the intestinal microbiota of children with ASD. They observed that *Oscillospira* increased significantly. In a study of depressed-like mice induced by chronic unpredictable mild stress, the stress induced an increase in the abundance of *Oscillospira;* treatment with *Sophora alopecuroides* (Leguminosae) derived alkaloids reduced the genus abundance (Cao et al., 2020). Johnson (2020) showed that social competence (a comprehensive measure of participants' extraversion, social skills, and communication ability) was highly correlated with *Akkermansia, Lactococcus*, and *Oscillospira*, which were more abundant in individuals with higher social scores. Similarly, the specific correlation between *Oscillospira* and *Ruminococcus* may indicate two independent microbes trying to colonize the microbial network of the AT group. Still, only one species can exert positive effects on patients with schizophrenia. Therefore, the existence of *Oscillospira* might become a “driver microbe” to prevent the colonization of harmful bacteria.

Furthermore, we evaluated the influence of, and changes in, various clinical characteristics on the colonization of *Ruminococcu*s before treatment. This study considered the multicollinearity problem between variables and utilized an all-subset regression to select the clinical variables to simplify the model. In the generalized linear model constructed in our study, the effects of nutrient intake and dietary habits were significant, and the regression coefficients were both positive. This indicates that levels of *Ruminococcus* colonization would decrease as the patients' flavors become weak and vegetables and fruit intake increases. Dietary composition and nutritional status are considered the most important factors in regulating the intestinal microbiota and have been proven as potential new therapies for many neuropsychiatric diseases (Sandhu et al., 2017). Hence, these results provide important and valuable suggestions for future clinical treatment.

Finally, it is feasible to establish animal models by recolonizing the gut of SPF mice with fecal microbiota from patients with SCZ and observing their behavior (Liang et al., 2019; Zhu et al., 2020a). Hyperlocomotion is typical of SCZ-positive symptoms (Akosman et al., 2021). In the open field test, the shorter distance traveled by AT mice than BT mice suggested a decrease in psychomotor hyperactivity in AT mice. Impairment of memory, sociability, and social novelty in SCZ mice, reflects impaired cognitive function and social interaction in SCZ patients (Liu et al., 2017). The memory ability to a familiar object, sociability, and social novelty was seen to be increased in AT mice, demonstrating that negative schizophrenia-like activity was ameliorated. It is notable that, although anxiety-like and depressive-like activity were often the early symptoms of schizophrenia (Lee et al., 2018), the time in open areas and the immobility time are not different in the two mice groups. Similar to our results, a recent study conducted by Zhu et al. did not find anxiety or depressive-like behaviors in mice receiving SCZ microbiota. Moreover, our patients were treated in the hospital for 2 weeks, which may have made these behaviors relatively insensitive to the patients' microbiota. Therefore, these data observed in SPF acceptor mice still provide further evidence for the gut microbes linked to SCZ severity and symptom changes in varying degrees.

In this study, the species composition and structure before and after the treatment were determined by 16S rRNA analysis. The relationship between changes in the intestinal flora and symptom improvement in SCZ patients was preliminarily verified by FMT. However, it is still necessary to provide more evidence to support this conclusion, particularly to reveal the mechanism beyond fecal bacterial transplantation. At the same time, it remains to be confirmed whether there is a causal interaction between changes in the microflora network and differences in symptoms. In addition, the sample size of the participants was small (only 25 patients), and it would be better to have a larger sample size in future studies.

## Conclusion

These results demonstrated that gut microbiota distributions differ when comparing the fecal samples of patients with SCZ after treatment to before treatment in the hospital. Host antipsychotic treatment could modulate SCZ symptoms by suppressing harmful bacteria and adjusting the assembly structure. The sensitive and well-connected species with marked changes, which might be closely related to decreased schizophrenia severity under antipsychotic treatment, could be considered potential species for future microbiota-directed therapies.

## Data Availability Statement

The datasets presented in this study can be found in online repositories. The names of the repository/repositories and accession number(s) can be found at: https://www.ncbi.nlm.nih.gov/, PRJNA821673.

## Ethics Statement

The human experiment was approved by the Medical Ethics Committee of The Fourth People's Hospital of Ya'an (approval number: 2021-1). The patients/participants provided their written informed consent to participate in this study. The animal study was reviewed and approved by All animal experiment procedures were approved by the Institutional Animal Care and Use Committee of the Sichuan Agricultural University (approval number: SYXKchuan2019-187).

## Author Contributions

MX, LZ, DaosP, TZ, and GW managed project and data. MX, DW, XW, and KZ recruited subjects and saved data. FL, XM, HY, DaoqP, and YiZ collected feces and performed the experiments LZ, LC, YoZ, ZS, DaosP, and GW analyzed and interpreted the results. LZ, MX, and DaosP prepared the draft. LZ, TZ, and GW revised and edited the manuscript. All authors read and approved the final manuscript.

## Funding

The present study was supported by Sichuan Science and Technology Program (2021YJ0166), which provided funding support for sample collection, parameters determination, animal purchase, and open access publication fees.

## Conflict of Interest

The authors declare that the research was conducted in the absence of any commercial or financial relationships that could be construed as a potential conflict of interest.

## Publisher's Note

All claims expressed in this article are solely those of the authors and do not necessarily represent those of their affiliated organizations, or those of the publisher, the editors and the reviewers. Any product that may be evaluated in this article, or claim that may be made by its manufacturer, is not guaranteed or endorsed by the publisher.

## Supplementary Material

16S rRNA sequencing reads have uploaded to NCBI. The accession code of sequence reads Archive in the National Center for Biotechnology Information (NCBI) BioProject database: PRJNA821673.
