# Supplementary material for: Intestinal Microbes in Patients With Schizophrenia Undergoing Short-Term Treatment: Core Species Identification Based on Co-Occurrence Networks and Regression Analysis
Source: Front Microbiol. 2022 Jun 17;13:909729. doi: 10.3389/fmicb.2022.909729 (PMC9247572; doi:10.3389/fmicb.2022.909729)
Supplement: Supplementary file 1 [file Data_Sheet_1.docx]

**Supplementary**

**Table S1.** Detailed clinical characteristics of subjects.

| Variables | Subjects | Variables | | Subjects |
| --- | --- | --- | --- | --- |
| Sample Size | 25 | Flavors (L/M/S) | 1/15/9 | |
| Sex (M/F) | 25/0 | F&V (N/O/U/E) | 0/13/12/0 | |
| Age (year) | 47.88 ± 11.00 | Iodine (N/O/U/E) | 0/18/7/0 | |
| Height (cm) | 166.96 ± 5.54 | Alcohol (N/O/U/E) | 15/7/2/1 | |
| Weight (kg) | 69.00 ± 10.56 | Smoking (N/O/U/E) | 9/2/1/13 | |
| Education (year) | 8.84 ± 3.05 | C&T (N/O/U/E) | 18/7/0/0 | |
| Medication (Y/N) | 25/0 | Sampling Interval (day) | 17.32 ± 1.60 | |

The values were expressed as means ± standard deviation.

M/F, male/female; Y/N, yes/no; L/M/S, light/medium/salty; F&V, fruits and vegetables; N/O/U/E, never/occasionally/usually/everyday; C&T, coffee and tea

**Table S2.** Changed scales score in before and after group.

| Scales | BT (n=25) | AT (n=25) | P values^1^ |
| --- | --- | --- | --- |
| BPRS | 52.00±7.67 | 46.80 ± 9.39 | 0.019 |
| SAPS | 36.04±22.99 | 24.96 ± 18.47 | 0.011 |
| SANS | 64.36±16.73 | 53.28 ± 24.61 | 0.0001 |

^1^ Paired T test. The results were expressed as means ± standard deviation.

BT, before treatment; AT, after treatment; BPRS, Brief Psychiatric Rating Scale; SAPS, Scale for Assessment of Positive Symptoms; SANS Scale for Assessment of Negative Symptoms

|  |
| --- |
